# Additive effects of the herbicide glyphosate and elevated temperature on the branched coral *Acropora formosa* in Nha Trang, Vietnam

**DOI:** 10.1007/s11356-016-8320-7

**Published:** 2017-01-22

**Authors:** C. Amid, M. Olstedt, J. S. Gunnarsson, H. Le Lan, H. Tran Thi Minh, P. J. Van den Brink, M. Hellström, M. Tedengren

**Affiliations:** 1 Department of Ecology, Environment and Plant Sciences (DEEP), Stockholm University, 106 91 Stockholm, Sweden; 2 Institute of Oceanography (IO), Nha Trang, Vietnam; 3 Department of Aquatic Ecology and Water Quality Management, Wageningen University, P.O. Box 47, 6700 AA Wageningen, The Netherlands; 4 Alterra, Wageningen University and Research Centre, P.O. Box 47, 6700 AA Wageningen, The Netherlands

**Keywords:** Coral bleaching, Climate change, Global warming, Pesticides, Digital image analysis, Chlorophyll, Zooxanthellae, Genotype, Tolerance, Adaptation

## Abstract

The combined effects of the herbicide glyphosate and elevated temperature were studied on the tropical staghorn coral *Acropora formosa*, in Nha Trang bay, Vietnam. The corals were collected from two different reefs, one close to a polluted fish farm and one in a marine-protected area (MPA). In the laboratory, branches of the corals were exposed to the herbicide glyphosate at ambient (28 °C) and at 3 °C elevated water temperatures (31 °C). Effects of herbicide and elevated temperature were studied on coral bleaching using photography and digital image analysis (new colorimetric method developed here based on grayscale), chlorophyll *a analysis*, and symbiotic dinoflagellate (*Symbiodinium*, referred to as zooxanthellae) counts. All corals from the MPA started to bleach in the laboratory before they were exposed to the treatments, indicating that they were very sensitive, as opposed to the corals collected from the more polluted site, which were more tolerant and showed no bleaching response to temperature increase or herbicide alone. However, the combined exposure to the stressors resulted in significant loss of color, proportional to loss in chlorophyll *a* and zooxanthellae. The difference in sensitivity of the corals collected from the polluted site versus the MPA site could be explained by different symbiont types: the resilient type C3u and the stress-sensitive types C21 and C23, respectively. The additive effect of elevated temperatures and herbicides adds further weight to the notion that the bleaching of coral reefs is accelerated in the presence of multiple stressors. These results suggest that the corals in Nha Trang bay have adapted to the ongoing pollution to become more tolerant to anthropogenic stressors, and that multiple stressors hamper this resilience. The loss of color and decrease of chlorophyll *a* suggest that bleaching is related to concentration of chloro-pigments. The colorimetric method could be further fine-tuned and used as a precise, non-intrusive tool for monitoring coral bleaching in situ.

## Introduction

Coral reefs are subject to increasing anthropogenic pressures, which has led to a circum-tropical decline of reef communities (Wilkinson, 2008). Declines are caused by overfishing (Pandolfi et al. 2003), pollution (e.g., Cervino et al. 2003, Fabricius 2005, D’Angelo and Wiedenmann 2014), and pesticide use malpractice (Van Hoi et al. 2009). Other major stressors causing coral decline and mass mortalities are caused by reduced salinity, shifts of dissolved inorganic nutrients (Goreau 1964, Coles 1992, Wiedenmann et al. 2013, D’Angelo and Wiedenmann 2014), and increased sea surface temperatures (Siebeck et al. 2006, Hoegh-Guldberg et al. 2007, Wooldridge 2009, Wooldridge and Done 2009) which have caused severe bleaching resulting in reduced coral reef abundance and diversity (Gardner et al. 2003, Baker et al. 2008, Van Hooidonk et al. 2013, Ban et al. 2014, Logan et al. 2014). Corals live in a close nutritional partnership with a wide range of endosymbiotic algae (*Symbiodinium*) commonly referred to as zooxanthellae. When exposed to severe stress, the zooxanthellae leave their coral host, which can lead to starvation and death (Weis et al. 2008). The corals turn pale and the phenomenon is referred to as “bleaching.”

Bleaching is considered an ecologically significant response variable for a number of reasons. Coral color has been found to be proportional to pigment content, e.g., chlorophyll *a*, which in turn is regulated by zooxanthellae densities (Winters et al. 2009). This relationship makes it possible to estimate degrees of stress by colorimetric methods measuring the loss of color. Moreover, proportional relationships have been found between zooxanthellae densities and coral tissue biomass (Porter et al. 1989, Szmant and Gassman 1990, Fitt et al. 1993), suggesting that coral fitness is directly related to zooxanthellae densities (however, see Cunning and Baker 2013). This notion of coral fitness is corroborated by the fact that symbiotic zooxanthellae (*Symbiodinium* spp.) supply the great majority (>95%) of coral energy demand (Muscatine and Porter 1977, reviewed in Rädecker et al. 2015) and therefore bleaching, as a sublethal stress response, is highly influenced by chlorophyll *a* content and zooxanthellae densities (Winters et al. 2009).

Many studies have quantified the interactive effects of increased temperature and other anthropogenic stressors (e.g., marine pollution through riverine discharges) on coral bleaching (review in Ban et al. 2014). However, only a few of these have examined the combined effects of temperature and herbicides on coral bleaching (Negri and Hoogenboom 2011, Negri et al. 2011). Understanding combined effects of stressors are especially important since reef building corals in tropical and subtropical waters already exist in close proximity of their upper thermal limits (Hoegh-Guldberg et al. 2007) and are known to be sensitive to numerous pollutants (Fabricius 2005, Ban et al. 2014, D’Angelo and Wiedenmann 2014).

In this study, bleaching was measured to assess the possible additive effects of an herbicide and increased temperature on the staghorn coral *Acropora formosa. A. formosa* is a key reef builder, common throughout the Indo-Pacific (Veron 2000). Indicators describing photosynthetic capacity, i.e. chlorophyll *a* content and zooxanthellae densities, were measured to quantify coral stress responses. In addition to these measurements, a new customized colorimetric method based on photography and digital image analysis in grayscale was developed to assess degrees of coral bleaching. The advantage of using of non-intrusive techniques, i.e. the presented colorimetric method, to quantify and detect early stages of coral stress is addressed and their potential use in future monitoring programs is discussed.

Glyphosate was chosen since it was found to be one of the most commonly used herbicides in rice cultures in the Nha Trang area, central Vietnam (based on interviews in nearby villages). Though glyphosate is used globally (Sirinathsinghji and Ho 2012), knowledge of its toxicological properties is scarce especially in marine organisms (Tsui and Chu 2003). Glyphosate has a high solubility in water (Solomon and Thompson 2003, Sirinathsinghji and Ho 2012), which increases its transport and bioavailability and puts aquatic organisms at risk (Sirinathsinghji and Ho 2012). Glyphosate (*N*-[phosphonomethyl]glycine; CAS registry number 1071-83-6) is a post-emergence, non-selective, broad-spectrum herbicide (Solomon and Thompson 2003). Its mode of action is inhibition of an essential enzyme (5-enolpyruvyl shikimate-3-P synthetase) needed for the synthesis of aromatic amino acids in plants (Devine et al. 1993). Its sublethal effects are characterized by chlorosis and decreased synthesis of aromatic amino acids and of the plant hormone indolic acetic acid. These toxicity symptoms lead ultimately to death through cessation of growth and necrosis (Solomon and Thompson 2003).

Representative coral colonies were collected in Nha Trang bay (Khanh Hoa Province, South Central Vietnam), a marine-protected area since 2001, with nine islands covering approximately 16,000 ha (O’Callaghan 2008). Despite the protective measures, pollution through riverine discharges and an increase of aquaculture has had deleterious effects on key species in the bay, i.e., fish, reef building corals, and other invertebrates (O’Callaghan 2008, Thu et al. 2008). In this study, the combined effects of the herbicide glyphosate and elevated temperature were investigated on *A. formosa* using a new digital image method together with pigment and zooxanthellae analyses. The main novelties of the present study are (1) the study of interactive effects of temperature and herbicide in controlled laboratory experiments, (2) the development of a new digital image analysis method and its comparison to chlorophyll *a* and zooxanthellae content, and (3) the comparison of stressor effects on two different types of zooxanthellae in *A. formosa*, collected from a polluted and a pristine area.

## Materials and methods

### Experimental design and timeline

**Fig. 1:**
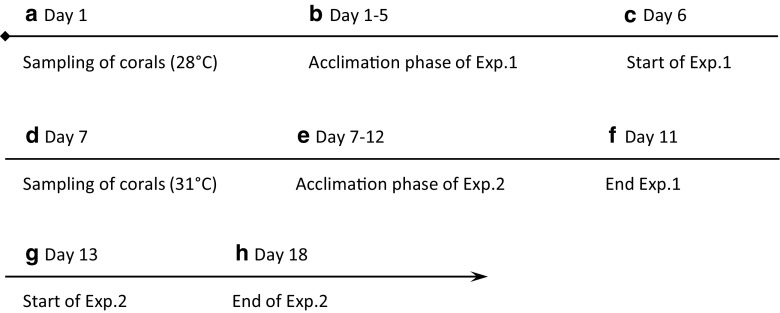
Experimental time-line in days. From day 1 (Nov. 2, 2012) to day 18 (Nov. 20, 2012). Exp. 1: Experiment 1 conducted at 28 °C. Exp. 2: Experiment 2 conducted at 31 °C

A two-factorial experiment design was used with “temperature” (two levels: low 28 °C and high 31 °C) and “herbicide exposure” (five levels: 0.0, 0.12, 1.2, 6.0, and 12.0 mg glyphosate L^−1^) as fixed factors and “coral stress responses”: (1) chlorophyll *a* content, (2) zooxanthellae densities, and (3) degree of bleaching as dependent variables. A time-line of the sampling and experiments is presented in Fig. 1.

### Collection of corals and seawater

**Fig. 2:**
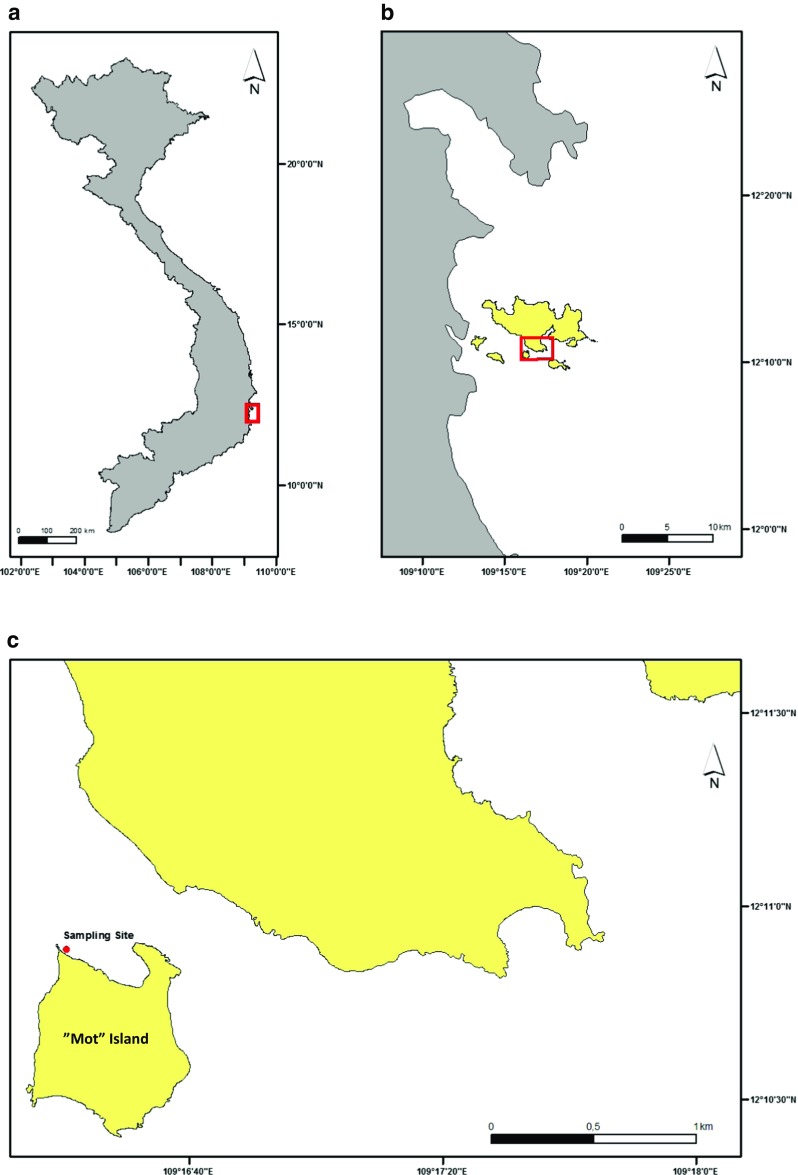
Vietnam is outlined and the city of Nha Trang (*red box*) indicated (**a**). The bay of Nha Trang, showing research area (*red box*) in more detail (**b**). The sampling site from where coral fragments, that w﻿ere subjected to treatments, were collected (**c**)

Coral fragments of the branched staghorn coral *A. formosa* were collected by SCUBA diving at 2.5 m depth during lowest astronomical tide, NW of “Mot” Island in the vicinity of Nha Trang Bay, Vietnam (12°10.911′N, 109°16.330′E; Fig. 2) on November 2 and 9, 2012. Coral branches were cut using a stainless steel plier and placed directly under water in 50 mL Sarstedt Falcon tubes (one fragment per tube), filled with seawater from the sampling site. Coral branches were kept individually to prevent possible chemical stress signals between fragments. The Falcon tubes were transported to the lab (ca 30 min) in a water-filled cooler (kept at ambient sea temperature). Seawater in the tubes was also replaced every 15 min to supply coral fragments with oxygenated sampling-site water and decrease stress and defense mechanisms (e.g. production of mucus). In addition, about 120 L of seawater was collected from the sampling site for each experiment and transported using 20 L plastic carboys to the lab. Collected seawater was kept dark to minimize photosynthetic activity and stored at ambient in situ temperature (28 °C) until the start of the experiments. In total, 70 coral branches of the same colony (80–90 mm terminal length) were collected, for experiments 1 and 2 (*n* = 27 per experiment) hereafter referred to as “Exp. 1” and “Exp. 2.” Additional fragments (*n* = 8·Exp^−1^) were collected for initial controls (T_0_ control), i.e. to observe initial conditions of the coral just after sampling (*n* = 4) and after the acclimatization phase in the laboratory before the start of the experiments (*n* = 4).

At the start of this study, a pilot experiment was conducted using coral branches sampled from a more pristine site located near “Mon” Island, 3 km SE of our current sampling site (“Mot” Island, see Fig. 2). However, the coral branches from Mon Island (*n* = 70 in total) were severely stressed after 72 h of acclimatization and were completely or partially bleached already at start of the exposures and could not be included in the exposure experiment, although their zooxanthellae composition was assessed as will be discussed later.

### Experimental setup and acclimatization phase

**Fig. 3:**
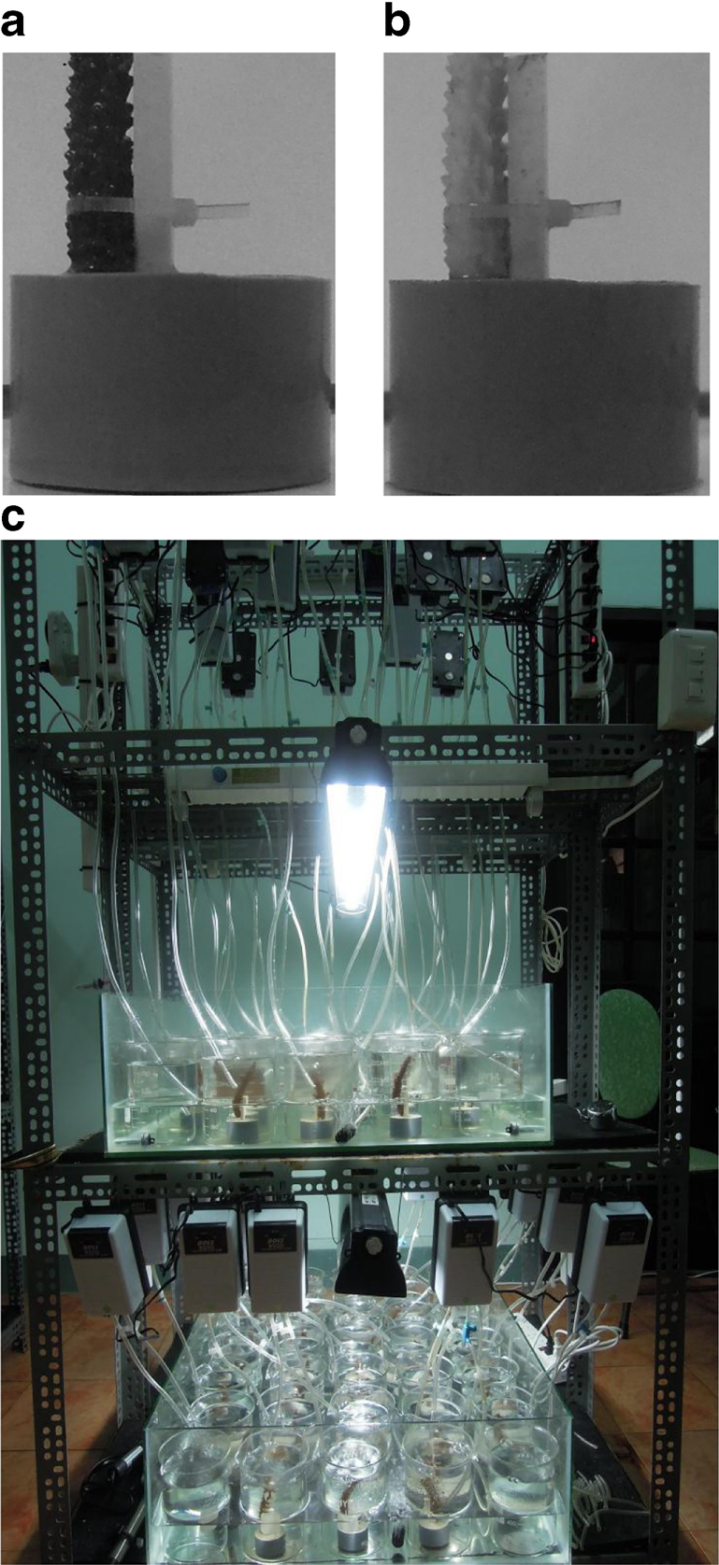
Experimental setup. Custom-made coral stand with a coral fragment before (**a**) and after exposure (**b**). Picture of experimental design (**c**)

Seawater used for each experiment was filtered through 1.2 μm glass fiber filters (Advantec GB-100R) to eliminate “larger” planktonic species possibly biasing exposure results. Small coral stands were made to hold up each coral branch during the experiments (Fig. 3a, b). The coral stands consisted of a base made of concrete (50 mm diameter, 25 mm height) in which a cut-off (70 mm) plastic chopstick was inserted before the cement had hardened. The coral branches were then tied in a vertical position to the chopsticks using plastic transparent zip ties (one branch per stand). Each mounted coral branch was then placed in an acid-rinsed (10% HNO_3_) 1 L beaker filled with 700–800 mL of filtered sea water (FSW). The beakers were randomly placed in a temperature-controlled water bath (a large custom-made aquarium L88 × W65 × H22 cm) to ensure a constant temperature of each replicate beaker during the experiments (Fig. 3c). Two water baths were used, one for the 28 °C treatment and one for the 31 °C treatment (see below). Air pumps were used to circulate the water of the water bath in order to keep its temperature homogenous. A diurnal 12:12 h light cycle was maintained using 30 W of aquaria lamps with continuous light regulated with timers. Light and temperature were monitored continuously during the experiments, every 10 min, using data loggers (Onset HOBO) to detect possible light and temperature deviations. Constant aeration to each beaker was provided using air pumps connected to Pasteur pipettes (one per beaker). Prior to the exposure period, coral fragments were acclimatized to laboratory conditions for 5 days, during which they were fed once with 100–250 individuals·mL^−1^ of newly hatched crustaceans (*Artemia* sp.) on day 3.

### Herbicide treatment

Coral fragments were exposed to the herbicide glyphosate (C_3_H_8_NO_5_P; CAS number 1071-83-6) purchased in Nha Trang under the common trade name “Clowdup 480SC.” The experiments were carried out at the Institute of Oceanography (IO), Nha Trang between November 8 and 13, 2012 (Exp. 1) and November 15 and 20, 2012 (Exp. 2) for an exposure period of approximately 5 days (110 h). Nominal exposure concentrations were 0.0, 0.12, 1.2, 6.0, and 12.0 mg·L^−1^ of glyphosate in the formulation, prepared from a stock solution of 120 mg L^−1^ (Exp. 1; prepared 21 Oct. 2012) and from a stock solution of 480 mg L^−1^ (Exp. 2; prepared 15 Nov. 2012). The experimental concentrations were obtained through a dilution series with experimental FSW. All concentrations in the paper are expressed as nominal concentrations of the commercial formulation. A subsample of the stock solution was brought to Sweden for confirmatory analysis and was analyzed at the Swedish University of Agricultural Science (SLU) using HPLC/tandem MS according to Hanke et al. (2008) and Jansson and Kreuger (2010) (method OMK 59). The confirmatory analysis showed that the actual concentration of the stock solution was 108 mg L^−1^. Thus, for actual concentrations, the nominal concentrations presented in this study should be multiplied by a factor of 0.9.

### Temperature treatment

Two experiments were carried out in series at different temperature. Exp. 1 was conducted at ambient sea temperature (28 °C) with five replicate beakers per glyphosate exposure and 7 replicates for the negative controls (i.e. no glyphosate added). Experiment 1 and experiment 2 were identical with the exception that they were conducted in 28 and 31 °C, respectively. The 3 °C difference between the experiments was chosen to correspond to a global warming temperature increase between a low CO_2_ emission scenario (+1.8 °C) and a high CO_2_ emission scenario (+2.4 to 6.4 °C) proposed by the Intergovernmental Panel on Climate Change (referred to in Hoegh-Guldberg et al. 2007). The temperature treatments were conducted in two successive experiments due to logistical constraints. In each experiment, the water temperature was obtained using two aquaria heaters placed in the water bath. Potential temperature gradients were avoided by continuously circulating the water baths with air pumps.

### Measured endpoints

Temperature and light conditions were continuously measured during the experiments using light and temperature loggers (UA-002-08 Onset, HOBO). Salinity (in PSU) was measured pre- and post-exposure to monitor possible deviations due to evaporation. Salinity was measured using a multimeter (WTW Multi 340i). The genotypes of zooxanthellae clades were confirmed post-exposure using Sanger sequencing of the ITS2rDNA region (see below). The following response endpoints to glyphosate and temperature treatments were determined: (1) bleaching degree (loss of color) of the corals measured by digital image analysis, (2) chlorophyll *a* content, and (3) number and cell division frequency of the zooxanthellae.

### Analysis of bleaching degree using digital image analysis

A new digital image analysis method was developed in this study for the quantification of bleaching degree. Pictures of each coral branch were taken in the laboratory before and after exposure (November 8 and November 13, Exp. 1; November 15 and November 20, Exp. 2) with a Nikon COOLPIX P300 digital camera (high resolution 24-bit JPEGs). Each coral branch was removed from its beaker and photographed in air in the laboratory. Laboratory windows were covered with black plastic bags and fluorescent ceiling lights were used as exposure light in order to reduce effects of different light conditions according to Thieberger et al. (1995) and Edmunds et al. (2003). Coral fragments attached to their stands were placed in a small photo-studio, i.e., in front of a white background consisting of four laminated perpendicular A4 sheets. Distance between camera and fragments was constant. Camera settings were set to auto. Corals were centered on a crosshair mark on the bottom sheet. Four perpendicular views were photographed of each coral branch, i.e. four images per coral replicate were photographed in order to get a good representation of the bleaching degree.

**Fig. 4:**
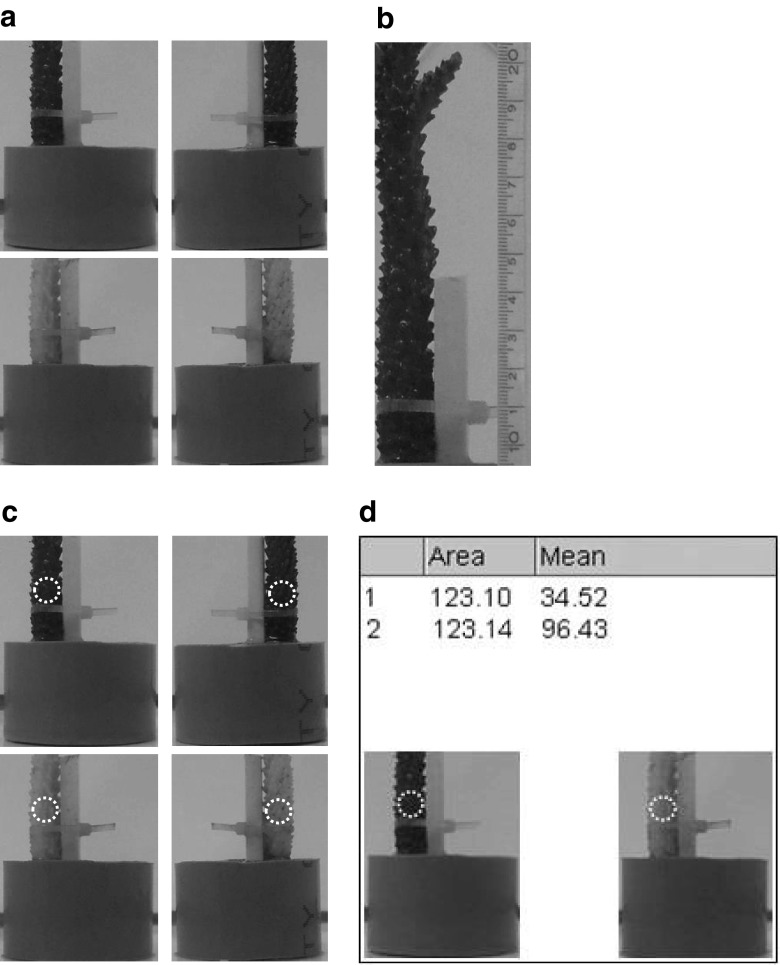
Image analysis of bleaching degree. Two opposite and perpendicular views of the same coral branch before (*upper images*) and after (*lower images*) exposure (**a**). Images were calibrated and integrated to a measuring scale to express areal coverage in mm^2^ (**b**). *Circles* (*white dashed line*) of approximately 123 mm^2^ were super imposed above bundle ties to quantify the bleaching intensity within the circle in two perpendicular views per coral fragment before and after exposure (**c**). Mean intensity of gray within *circles* (range 0–255) is calculated in one of the perpendicular views before (*1*) and after (*2*) exposure (**d**). Note the significant change in mean intensity of gray before and after exposure, i.e. bleaching severity is correlated to higher pixel values. A value of 255 is equivalent to 100% saturation (proportion of gray) or brightness (lightness and darkness)

Pictures were analyzed digitally using ImageJ (version 1.46r). Pictures were converted from 24-bit color to 8-bit grayscale (pixel range 0–255) in order to reduce variability associated with auto-settings, i.e. lens focal length (distance to focus), aperture speed (light shutter speed), and aperture size (size of shutter opening). Two of the four opposite views (second and fourth) were analyzed for each coral fragment before and after exposure (Fig. 4a). All images were calibrated to a measuring scale (Fig. 4b). A circle was superimposed on each coral branch picture just above the bundle ties (Fig. 4c), and only pixel values within the circle were analyzed. The circle area was approximately 123 mm^2^. The same circle area corresponded to the coral surface area that was extracted for chlorophyll *a* and zooxanthellae measurements (see below). The arithmetic mean of pixel values (range 0–255) in each circle was calculated (Fig. 4d) and used to obtain a combined mean value of the second and fourth perpendicular view. This value was called mean intensity of gray (MIG). One MIG value per replicate before and after exposure was compared using the following function:

MIG_RE_ = 1 − (MIG_S_ / MIG_E_).

where MIG_RE_ is the relative mean intensity of gray; MIG_S_ is the mean intensity of gray at start, i.e. before exposure; and MIG_E_ is the mean intensity of gray at the end, i.e., after exposure.

### Chlorophyll *a* and zooxanthellae

Coral tissue samples were collected post-exposure from each replicate. Surface samples were consistently taken from the fourth perpendicular view (see above). Coral tissues were taken from a circle area of 123 mm^2^, corresponding to the circle used for the photometric measurements described above. A plastic lid of the right dimension (123 mm^2^) was held over the coral, above the bundle, and the coral tissue inside the circle was blasted off with 10 mL of FSW using 5 mL disposable syringes. The tissue homogenate was collected in Sarstedt Falcon tubes, and the procedure was repeated until all tissue within the circle was collected and only the coral skeleton was left. The collected tissue samples were allowed to settle in order to separate tissue from excreted mucus, which was floating on top. Thereafter, 2 mL aliquots were pipetted into 2 mL microtubes (Sarstedt). A 0.5 mL subsample was taken from each microtube for chlorophyll *a* analyses, and the rest was kept for zooxanthellae measurements. The 0.5 mL samples were filtered (Glass fiber Advantec GB-100R). The filtrate was discarded and the filters were placed in sterile Sarstedt microtubes and stored dark at −20 °C until extraction. Chlorophyll *a* was extracted in 90% acetone and measured on a fluorometer (Turner 10AU™) according to Welschmeyer (1994) and Suggett et al. (2010) without acid treatment.

The remaining 1.5 mL tissue samples were used for zooxanthellae analyses and were immediately preserved in 50% solution of glutaraldehyde. Two aliquots per replicate were stained with acidic Lugol solution, and the total number of zooxanthellae was microscopically (×400 magnification) determined using a Bürker counting chamber (10 replicates per count). All counts were done using blind analysis to avoid bias. Cells under division (i.e., cells undergoing mitosis) were counted as two cells in the total cell count. The number of mitotic cells was also recorded separately and was named mitotic index (MI). Chlorophyll *a* content was expressed as μg·mm^−2^, zooxanthellae and MI-cell counts were expressed as number·mm^−2^.

### Genotype determination of zooxanthellae clade

Tissue samples were collected from control fragments from Mot Island (*n* = 4·Exp^−1^) and from Mon Island (*n* = 7) and stored in 70% ethanol for genetic identification of the Symbiodinium. The methods followed Hellström (2011). Shortly, the ITS2rDNA region was amplified using the primers in LaJeunesse and Trench (2000) and a modified PCR protocol based on LaJeunesse and Trench (2000) and Porto et al. (2008). This region gives a resolution on three different taxonomic levels: clade, type, and subtype (Howells et al. 2016) differentiating subtypes with different resilience traits. The amplicons were directly sequenced and identified by BLAST search tools prior to alignment with published reference material in NCBI GeneBank.

### Statistical analysis

Treatment effects (temperature and glyphosate exposure) on six dependent variables (chlorophyll *a*, zooxanthellae counts, MI counts, MIG_E_, MIG_RE_, and salinity) were tested statistically using R (version 2.13.2). Variables were ln-transformed when necessary to fulfill assumptions of homoscedasticity and normality. Parametric assumptions were tested post analysis on standardized residuals. Each experiment consisted of 27 coral fragments, i.e. *n* = 7 for controls and *n* = 5 for each glyphosate concentration tested (0.12, 1.2, 6.0, 12.0 mg glyphosate·L^−1^).

Three separate analyses were conducted. First, the effect of herbicide exposure (five levels) and temperature (two levels) was evaluated using analysis of variance to compare any significant differences between combinations of treatment exposure (herbicide and/or temperature exposure) with experimental controls. Second, multiple linear regressions were conducted to examine possible regressions between chlorophyll *a* (dependent variable) and predictors, i.e. zooxanthellae counts, MI counts, MIG_E_, MIG_RE_, salinity, temperature, and glyphosate exposure. The Akaike information criteria (AIC) were used to find best regression models. Third, Pearson correlations and simple linear regressions were conducted to compare the relation between chlorophyll *a* and zooxanthellae found in this study to other colorimetrical and pigment relations reported in previous published studies. *α* was set at 0.05 in all tests.

## Results

### Light, temperature, and salinity measurements

Light was rather constant according to logger data, except for the variations between day and night set to a diurnal 12:12 h light cycle. Water temperature was $\overline{x}$ = 28.4 °C in Exp. 1 (*n* = 3365; SD = 0.4 °C) and $\overline{x}$ = 31.2 °C in Exp. 2 (*n* = 3365; SD = 0.3 °C). There were no significant differences between salinity pre- and post-exposure. The salinity was slightly higher in Exp. 2 (34.5 PSU) than in Exp. 1 (32.6 PSU) due to a higher evaporation with the 3 °C increase in temperature; see Table 1.

**Table 1:** Salinity measured post exposure

Exposure	Exp.	n	PSU ($\overline{x}$)	SD (in PSU)
Control	1	7	33.6	0.28
G_0.12	1	5	33.6	0.22
G_1.2	1	5	33.4	0.16
G_6.0	1	5	31.9	0.12
G_12.0	1	5	30.5	0.37
Control	2	7	35.0	0.68
G_0.12	2	5	35.1	0.41
G_1.2	2	5	34.6	0.54
G_6.0	2	5	34.1	0.80
G_12.0	2	5	34.0	0.48

Exposure describes tested glyphosate concentrations in mg L^−1^ with the exception of experimental controls. Exp. describes different temperature treatments where 1 is +28 °C and 2 is +31 °C. *n* is the number of replicates used. PSU values show mean and SD for each exposure group

### Digital image analysis

**Fig. 5:**
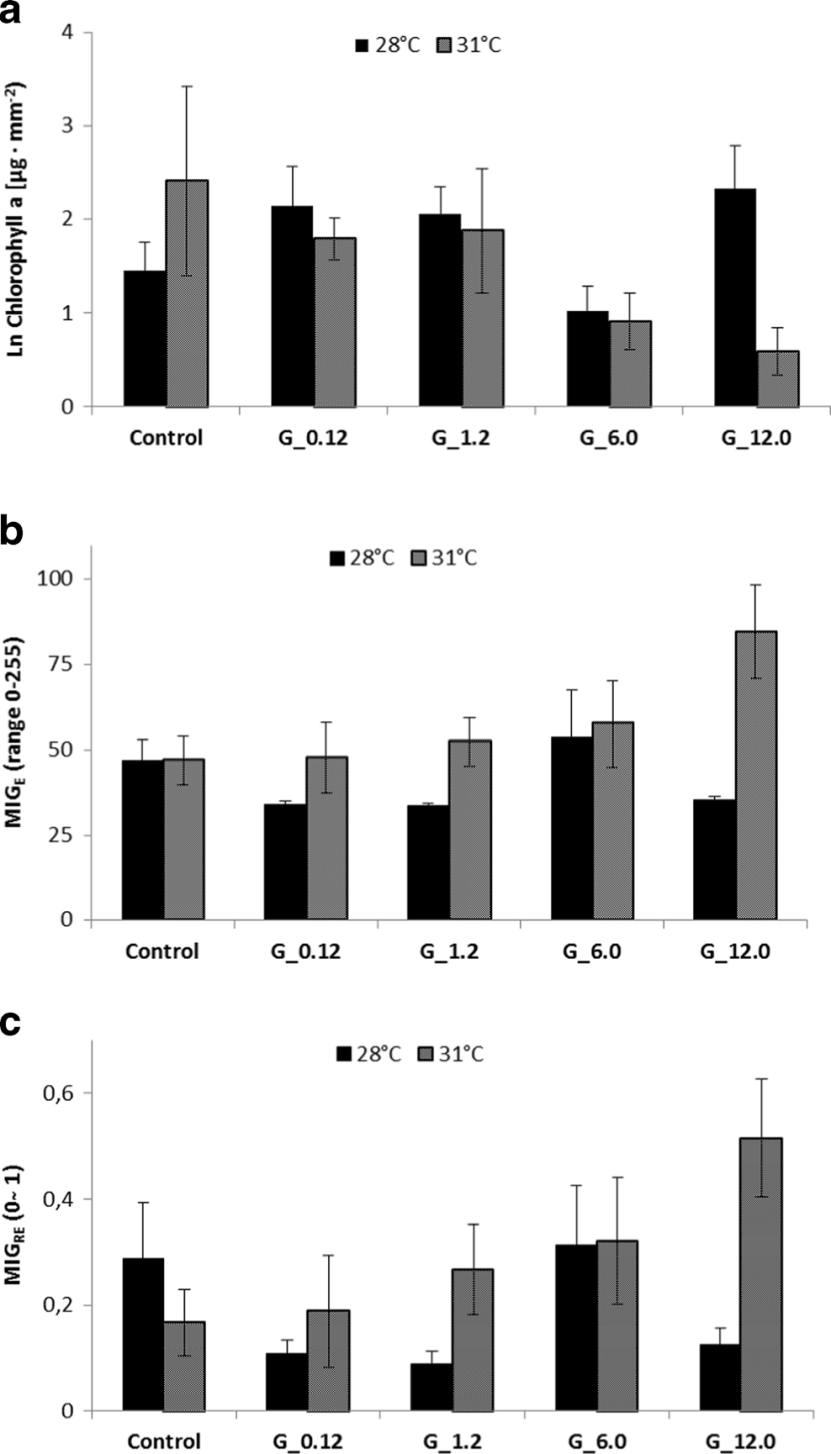
Effects of herbicide exposure at +28 and 31 °C on ln-transformed chlorophyll *a* content (**a**), for the photometrical variable MIG_E_ (**b**) and for the photometrical variable MIG_RE_ (**c**). The highest herbicide concentration (G_12.0) at the elevated temperature exposure (31 °C) differs significantly from controls for all three measured variables. No other combination of treatments differs significantly from experimental controls. *Error bars* are SE. In Fig. 4 **b**, **c**, values are proportional to bleaching degree

There was no significant treatment effect on coral pigmentation by temperature or glyphosate exposure alone. However, the interaction between temperature and glyphosate exposure was significantly different from the controls for the highest concentration of herbicide (12.0 mg L^−1^) at the increased temperature treatment for both MIG_E_ and MIG_RE_ (two-way ANOVA followed by Tukey HSD post hoc test, F_9, 44_ = 8.5, *P* < 0.01 and F_9, 44_ = 3.1, *P* < 0.01 respectively; Fig. 5b, c).

### Chlorophyll *a* and zooxanthellae measurements

Similar results were observed for the chlorophyll *a* variable, where significant interaction effects of temperature and glyphosate exposure were found for the highest herbicide concentration at the elevated temperature treatment (two-way ANOVA followed by Tukey HSD post hoc test, F_9, 44_ = 3.1, *P* < 0.001; Fig. 5a). No significant effects were observed for zooxanthellae or MI counts, independent of treatment combinations.

### Determination of the most influential predictor(s) of chlorophyll a

Multiple regression analyses were conducted according to the following model setup:

1. Chlorophyll *a* ~ Zooxanthellae counts + MI counts + MIG_E_ + salinity + temperature + exposure
2. Chlorophyll *a* ~ Zooxanthellae counts + MI counts + MIG_RE_ + salinity + temperature + exposure

**Fig. 6:**
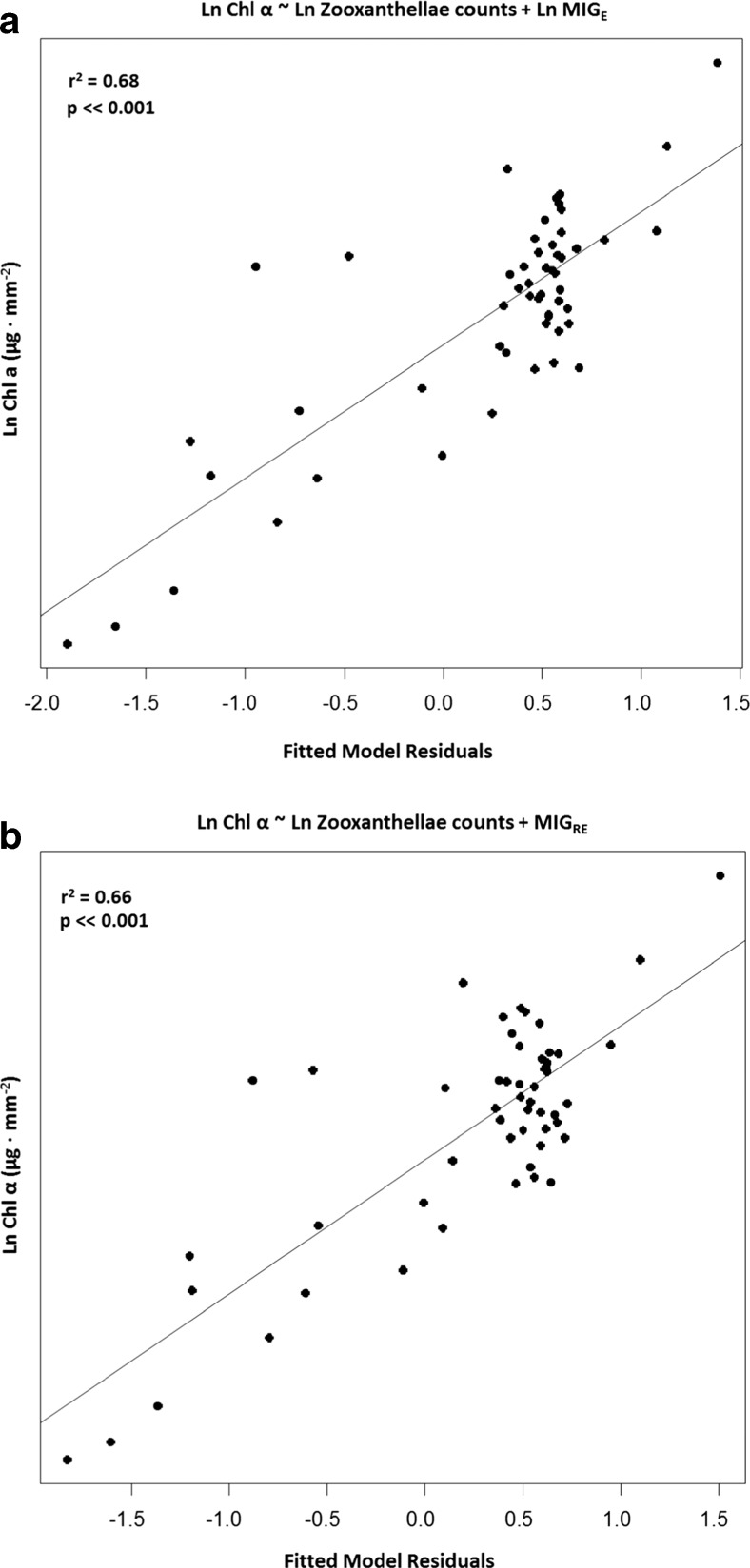
Plots from multiple regression analyses where the residuals for the best model, highly influenced by zooxanthellae densities and photometric variables, are plotted against chlorophyll *a*. Initial model inputs were identical with the exception of photometric variables, MIG_E_ in (**a**) and MIG_RE_ in (**b**)

In both cases, variations in chlorophyll *a* content were best described by zooxanthellae counts and the photometric variables (F_2, 51_ = 53.8, *P* < 0.001 zooxanthellae counts and MIG_E_ respectively, *r* ^2^ = 0.68 and F_2, 51_ = 49.3, *P* < 0.001 zooxanthellae counts and MIG_RE_ respectively, *r* ^2^ = 0.66). Results of the multiple regression models 1 and 2 are presented in Fig. 6a, b.

**Fig. 7:**
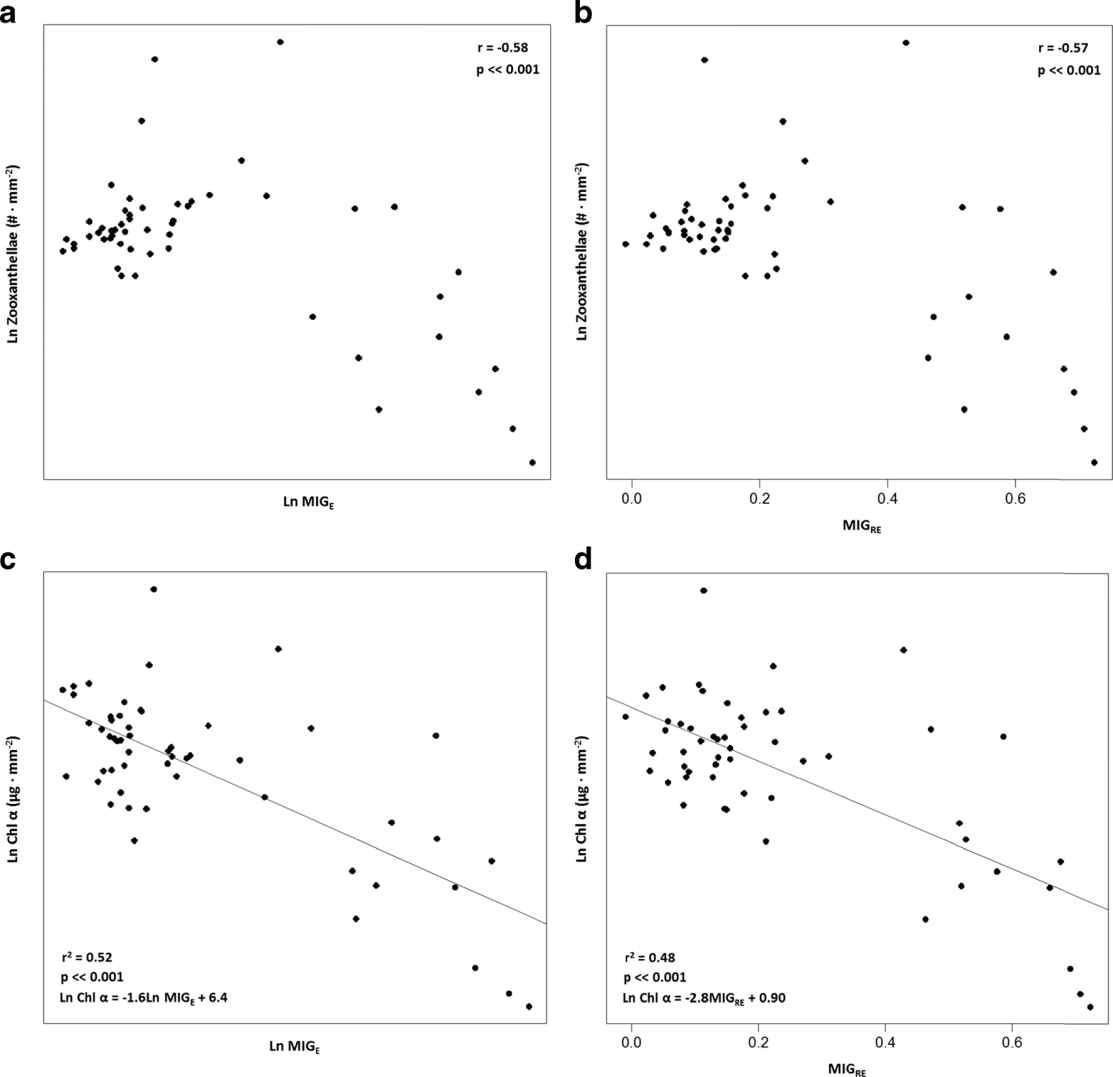
Significant correlations and regressions between the number of zooxanthellae or chlorophyll *a* content and photometric variables in both experiments, total *n* = 54 (**a**, **b** and **c**, **d**, respectively). Note the negative relationship describing decline in the number of zooxanthellae or chlorophyll *a* content with increasing MIG_E_ or MIG_RE_ values

The number of zooxanthellae correlated significantly with the photometric variables (Pearson correlations; *P* < 0.001 MIG_E_, *r* = −0.58 and *P* < 0.001 MIG_RE_, *r* = −0.57; Fig. 7a, b respectively). Simple linear regressions revealed significant relationships between chlorophyll *a* and photometric variables (MIG_E_, F_1, 52_ = 56.9, *P* < 0.001, *r* ^2^ = 0.52 and MIG_RE_, F_1, 52_ = 48.2, *P* < 0.001, *r* ^2^ = 0.48; Fig. 7c, d respectively).

### Genotype analysis

The *Symbiodinium* types in all fragments from Mot Island were identified as C3u (NCBI accession number GU111879) and from Mon Island as C21 and C23 (NCBI accession number AY239372 and EU499102)

## Discussion

Branches of the staghorn coral *A. formosa* were not significantly bleached by temperature increase (3 °C) or by herbicide exposure alone. However, the combination of these two stressors, i.e. both elevated water temperature and herbicide exposure, caused a significant decrease of chlorophyll *a* (Fig. 5a) and a significant color loss (Fig. 5b, c). The similar responses of the photometric variables and the chlorophyll *a* suggest that coral bleaching is related to the concentration of photopigments and moreover quantifiable by digital photometry methods. This is further supported by the significant regressions found between chlorophyll *a* and the two photo-variables MIG_E_ and MIG_RE_ (Fig. 7c, d).

Various environmental stressors, such as contaminants (Ban et al. 2014) and increased sea surface temperatures (Coles and Jokiel 1977, Brown 1997, Hoegh-Guldberg 1999, Van Hooidonk et al. 2013, Logan et al. 2014), can cause coral bleaching. Results from the present survey is in line with previous studies which highlight the importance of synergistic effect caused by rising temperatures in combination with pollutants (reviewed in Ban et al. 2014).

Predictive scenarios regarding elevated temperature and subsequently increased stress on coral communities are particularly important for reef building corals in tropical and subtropical waters. Coral reefs worldwide are already experiencing their thermal tolerance limits (Hoegh-Guldberg et al. 2007) and in many areas, including Vietnam, exposed to heavy pollution, including pesticide malpractice use (Van Hoi et al. 2009), through riverine discharges. On the other hand, predictive models of coral bleaching need to consider adaptive responses in the coral host (Palumbi et al. 2014) and stress tolerance differences in the symbiotic algae (Coles et al. 1976, Berkelmans 2002, Riegl et al. 2011).

The critical temperatures for activating coral bleaching differ considerably between geographic regions indicating that corals and their symbionts undergo adaptation to their environments (e.g. Coles et al. 1976, Berkelmans 2002, Riegl et al. 2011). Corals showing altered bleaching patterns may adapt to warming over time (Guest et al. 2012, Maynard et al. 2008, Pratchett et al. 2013). Resilient corals of the same species seem to acclimatize to increasing temperatures by hosting stress-tolerant zooxanthellae types (Hume et al. 2015, Silverstein et al. 2015, Howells et al. 2016) combined with adaptation of the coral itself (Barshis et al. 2013, Palumbi et al. 2014). These adaptations include regulations of an array of thermal response genes, including heat shock proteins and antioxidant enzymes (Barshis et al. 2013). However, multiple stressors may slow down this acclimatization mechanism causing more permanent damage to corals (Carilli et al. 2009).

These cellular mechanisms may explain why zooxanthellae densities in the Nha Trang bay corals, collected in the vicinity of Mot Island, did not differ significantly between temperature exposures. The endosymbiotic zooxanthellae clade in all these fragments was identified as C3u. In Vietnam, the clade C3u is found in areas with high chlorophyll *a* contents or close to human populations or fish farms with high nitrogen effluent (Hellström, 2011). The clades from the MPA corals in this study were identified as C21 and C23, which have been connected to more pristine sites. These results, i.e. that coral fragments from Mot Island endured the full length of the experimental period while bleaching rates were high during the acclimatization phase for coral branches sampled from Mon Island, are very interesting and indicate that the corals have different sensitivities and are able to evolve and adapt to anthropogenic stressors. More importantly, variability associated to zooxanthellae genotypes may reflect survival properties of coral species. In this study, the coral fragments collected from Mot Island were surrounded by fish farms and subjected to riverine discharges from Nha Trang. Mon Island on the other hand is isolated and less exposed to anthropogenic stress, i.e., aquaculture farms and riverine discharges. It is therefore likely that corals near Mot Island are more adapted to environmental stressors and are thus more tolerant than the ones at Mon Island. Results from this study show that the combined stress of elevated temperature and high herbicide exposure has more pronounced effects on coral bleaching than one of these stressors alone. This is in line with the study of Negri et al. (2011) where three herbicides (photosystem II inhibitors) with different modes of action were used simultaneously. Further studies are needed where the synergistic effects of herbicides with different modes of action is tested (Ban et al. 2014).

The essential implications of the present study, however, are described in the additive association of temperature to environmental stressors, e.g. herbicide exposure, but also in the revelation that there is a threshold even for well-adapted corals, e.g. corals with C3u zooxanthellae clades, to human-induced stress.

### Analysis of coral bleaching degree using digital photography

The digital photometry method developed in this study, i.e. by determining the mean intensity of gray variables MIG_E_ and MIG_RE_, was successful in detecting the bleaching degree of coral fragments in response to temperature and herbicide treatments (Fig. 5b, c). The fact that MIG_E_ and MIG_RE_ results were very similar to those expressed in chlorophyll *a* content and that variations in chlorophyll *a* were best described by the photometric variables and number of zooxanthellae, i.e. multiple linear regression results for model 1 and 2, respectively (Fig. 6a, b), adds further weight to the conclusion that there is a relationship between color, chlorophyll *a*, and the number of zooxanthellae as suggested by Winters et al. (2009). This relationship is described in the provided regression equations (Fig. 7c, d) where chlorophyll concentrations can be derived from digital imagery through the photometric variables MIG_E_ and MIG_RE_.

Despite that the bleaching analysis was performed in grayscale (range 0–255) to exclude variations associated to camera settings, better correlation and regression results were acquired compared to earlier studies performed in color (Fig. 7a–d) but also in comparison with a recent study performed in grayscale. In this study, the number of zooxanthellae correlated significantly with the photometric variables (*P* < 0.001, MIG_E_, *r* = −0.58 and MIG_RE_, *r* = −0.57). In Edmunds et al. (2003) the correlation between the number of zooxanthellae and the photometric variable was *r* = −0.45 (*P* < 0.001), where color values were based on three standard bands, i.e. red, green, and blue, compared to one band in this study, i.e. grayscale. Simple linear regressions revealed significant relationships between chlorophyll *a* and photometric variables (*P* < 0.001, MIG_E_, *r* ^2^ = 0.52 and MIG_RE_, *r* ^2^ = 0.48). In Siebeck et al. (2006), a significant relationship was found (*P* < 0.001, *r* ^2^ = 0.36) between chlorophyll *a* and color reference card values but the resolution of the color card method was low. Also, in comparison with a recent study performed in grayscale (Chow et al. 2016, *P* = 0.04, *r* ^2^ = 0.38), the photometric variables in this study rendered better results.

Our results with this new photometry method are promising; however, more work is needed to adapt the method to be used directly in the field by SCUBA diving. The advantages of taking pictures in air are excluded bias from variations in light intensity and focal distance. More approximate methods, e.g., the coral color reference card developed in Siebeck et al. (2006), were developed for field conditions and are inaccurate when higher resolutions are needed (Chow et al. 2016) and may be more useful in combination with photometric methods developed in this study. Photometric methods also reduce interobserver variability. The possibility to accurately detect coral community response to a myriad of stressors without the use of intrusive and time-consuming techniques has been the main incentive behind this relative new field of assessing physiological stress by means of color deviations.

### Variability associated with zooxanthellae densities

Alternatively, the lack of significant difference between treatment replicates and experimental controls could be accredited by the large natural variation of zooxanthellae densities, influenced by spatial orientation of coral fragments relative to sunlight, attenuation of light with depth and/or water turbidity. Moreover, zooxanthellae densities might differ vertically within coral fragments. During sampling, we observed irregular coloring (i.e. brightness of color hues) in the basal part of the fragments were tissue samples were taken. This was however not the case for the uppermost part (terminal 20–30 mm), which deviated much less between fragments. Finally, the lack of significant differences in zooxanthellae between treatments may also be due to the counting method used since Lugol does not discriminate between live and dead cells, which may have led to an overestimation of zooxanthellae counts in this study. A more suitable method would have been to estimate cell counts by means of a fluorometric method such as fluorescein isothiocyanate (FITC), which differentiates between live and dead cells. The mitotic index (MI), a division frequency of zooxanthellae undergoing mitosis, has been suggested as a potential stress measurement endpoint (Brown 1988). The MI has been suggested to increase in order to counteract the expulsion of zooxanthellae by the coral host following herbicide and/or temperature exposure (Hoegh-Guldberg and Smith 1989; Fitt et al. 1993; Jones 1995). No significant treatment effects were observed on the MI in this study. This further questions the relevance of MI as a stress response variable since it can both increase and decrease in response to stress (Jones 1997)*.*

## Conclusion

Coral bleaching is important to monitor since the symbiotic zooxanthellae (*Symbiodinium* spp.) supply the great majority (>95%) of the coral host energy demand (Hoegh-Guldberg et al. 2007). Most importantly, the proportionality of chlorophyll *a* content to color, which in turn is highly regulated by zooxanthellae densities (Winters et al. 2009), justifies the use of colorimetric methods to assess coral physiology. The results from this study show that there is a significant correlation between chlorophyll *a* and intensity of gray measured using digital photography analysis and that our colorimetric variables MIG_E_ and MIG_RE_ are good descriptors of coral bleaching and less intrusive and time consuming than chlorophyll *a* extraction and analysis. Based on our results, we also suggest that future steps should be taken to further develop photography and digital image analysis of coral bleaching in situ as a proxy for detecting physiological changes such as loss of chlorophyll and zooxanthellae.
